# Crystallographic Texture of the Mineral Matter in the Bivalve Shells of *Gryphaea dilatata* Sowerby, 1816

**DOI:** 10.3390/biology11091300

**Published:** 2022-08-31

**Authors:** Alexey Pakhnevich, Dmitry Nikolayev, Tatiana Lychagina

**Affiliations:** 1 Borissiak Paleontological Institute, Russian Academy of Sciences, 117647 Moscow, Russia; 2 Frank Laboratory of Neutron Physics, Joint Institute for Nuclear Research, 141980 Dubna, Russia

**Keywords:** *Gryphaea dilatata*, crystallographic texture, pole figures, neutron diffraction, recrystallization, thick-walled shells

## Abstract

**Simple Summary:**

A new for paleontology method has been applied to study the orientation distribution of the crystals that compose the fossils of mollusk shells. The method is based on the use of neutrons with high penetrating power and makes it possible to study bulk shells without destroying them. In this work, we studied how the habitat conditions and the process of fossilization influenced the distribution of shell crystallite orientations. It was possible to establish a relationship between the distribution of orientations and the shape of the shells.

**Abstract:**

It is assumed that the crystallographic texture of minerals in the shells of recent and fossil mollusks is very stable. To check this, it is necessary to examine the shells of animals that had lain in sediments for millions of years and lived in different conditions. It is revealed that the crystallographic texture of calcite in the shells of *Gryphaea dilatata* from deposits from the Middle Callovian–Lower Oxfordian (Jurassic), which lived in different water areas, is not affected by habitat conditions and the fossilization process. The crystallographic texture was studied using pole figures measured by neutron diffraction. The neutron diffraction method makes it possible to study the crystallographic texture in large samples—up to 100 cm^3^ in volume without destroying them. The recrystallization features of the *G. dilatata* valve, which affect the crystallographic texture, were discovered for the first time. This is determined from the isolines appearance of pole figures. The crystallographic texture of the *G. dilatata* mollusks’ different valves vary depending on their shape. The pole figures of calcite in the thick-walled valves of *G. dilatata*, *Pycnodonte mirabilis*, and *Ostrea edulis* are close to axial and display weak crystallographic texture.

## 1. Introduction

Bivalve mollusks from the family Gryphaeidae were a frequently occurring fauna element in the seas of the Middle and Late Jurassic of the East European Platform. The most common and numerous were the species of the genus *Gryphaea* Lamarck, 1801. The shells of these mollusks were large and thick-walled. At the same time, the left valve is strongly convex with a curved umbo, while the right one is flattened. Such a shell was necessary as a passive defense against predators (on 169 valves of *Gryphaea dilatata* Sowerby, 1816 from the Callovian deposits near the village of Sukhochevo (Oryol region, Russia), where only one trace of a predator attack was found); it well withstood abrasion in shallow water due to wave activity, and thickened walls coalesced better when jars were formed. The microstructure of gryphaea valves is shown through numerous layers of plates. Such a shell organization turned out to be very evolutionarily successful; therefore, the genus existed from the Late Triassic to the Paleogene [1]. A similar adaptation was also characteristic of the Cenozoic Gryphaeidae, and it also manifested itself within the closely-related bivalve family, Ostreidae. 

The shells of recent and fossil *Ostrea edulis* Linnaeus, 1758 (Ostreidae family), consist of calcite. The microstructures of the family Ostreidae representatives are well studied [2,3,4]. The crystallographic texture of calcite is also known for *O. edulis* valves [5]. Earlier, it was shown [6] that there is no direct connection between the type of microstructure and crystallographic texture, which is why we do not pay much attention to microstructure in the present study.

Crystallographic texture is a collection of orientations of a polycrystalline sample. The shells consist of calcium carbonate polycrystals and are characterized by the anisotropy of physical and mechanical properties, which is closely related to the preferred orientation (texture) of their grains. Texture is formed during shell growth and can be influenced by various environmental factors. Recently, an attempt has been made to study the effects of the elemental content of the shells of the bivalve mollusks, *Mytilus galloprovincialis* Lamarck, 1819, on their crystallographic texture [7]. 

Quantitative information about crystallographic texture is contained in measured pole figures, which are two-dimensional distributions of relative volumes for specific crystallographic directions on a unit sphere [8]. Values on a pole figure are given in mrd (multiple random distribution) units. The value 1 corresponds to the uniform isotropic distribution of crystalline orientations. Most pole figure measurements of shells are carried out by means of X-rays or electron backscatter diffraction (EBSD) [9,10,11,12,13,14,15]. However, these techniques are very local because of the low penetrating power of these radiations into matter. The much greater penetration depth of neutrons in the sample material allows for the investigation of centimeter size samples in transmission geometry, which yields non-destructive texture measurements. Moreover, X-ray or EBSD techniques allow us to study a relatively small part of a valve, while neutron diffraction allows us to measure almost the whole bulk shell [16]. Complete pole figures can be obtained without intensity corrections by means of neutron diffraction; the grain statistics are better, which is of particular interest for coarse-grained samples [17].

The arrangement of crystals in recent shells of *O. edulis* is not highly ordered, i.e., the texture is not too sharp—up to 2.53 mrd—compared to calcite from the shells of recent mussels, such as *M. galloprovincialis*—which are up to 12.53 mrd [18]. It should be emphasized that these numbers were obtained for the whole bulk shells. It can be suspected that the thickened shell of fossil mollusks may have a more ordered texture, which is necessary to preserve its integrity. In this regard, gryphaea shells were selected from three localities in order to solve several problems of the crystallographic texture evaluation of the mineral matter of these mollusks’ valves.

Previously, we compared the textures of calcite and aragonite in the shells of recent mussels and oysters from different parts of the areal and geologic ages, but only with a difference of 30 thousand years [18].

The purpose of this work is to compare the crystallographic texture of the valve’s mineral matter of the bivalve mollusks, *G. dilatata,* from three remote localities that have been formed under different diagenetic conditions. 

The objectives of the study included:

1. Determination of the crystallographic texture sharpness of the *G. dilatata* mineral substance;
2. Assessment of the influence of various habitat conditions and diagenetic processes on the texture;
3. Reveal the crystallographic texture dependence on the valve shape;
4. Reveal the texture similarity in two representatives of the Gryphaeidae family;
5. A comparison of recent and fossil valves of *O. edulis;*
6. Reveal the common features of the crystallographic texture in thick-walled shells.

## 2. Materials and Methods

The bivalve mollusk shells of *G. dilatata* were chosen as objects to study. This is a widespread species whose shells are found in large amounts in Jurassic deposits. Large samples of adult mollusks have been studied. They were measured one at a time since they contained sufficient substance for analysis. Both convex-left and flattened-right valves were studied.

**Figure 1:**
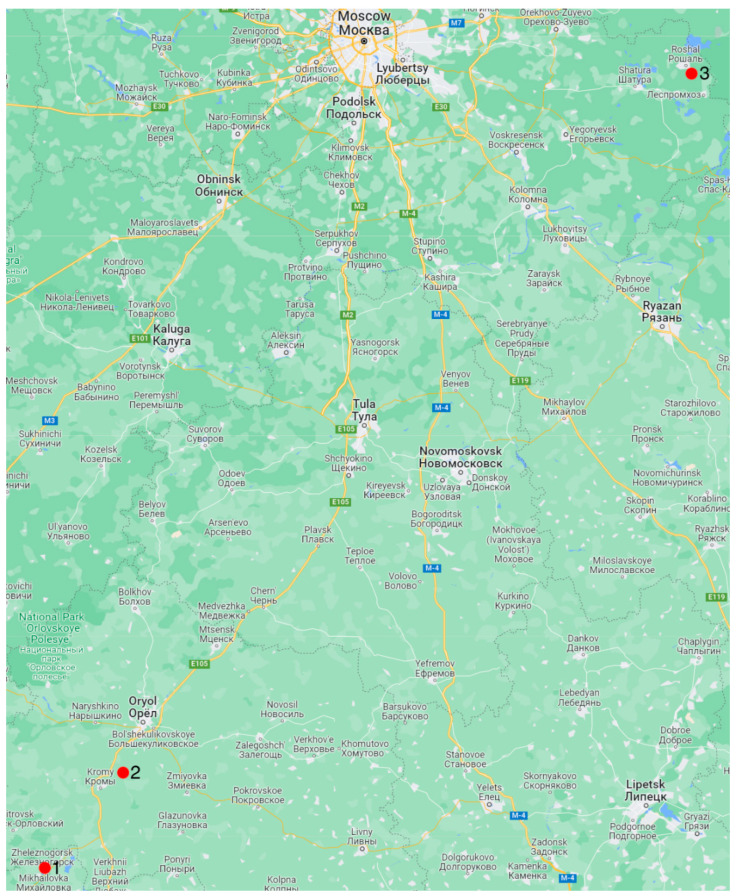
Collection locations of *Gryphaea dilatata* Sowerby, 1816: 1—Kursk region, near the town of Zheleznogorsk, Mikhailovsky quarry; 2—Oryol region, Kromy district, sand quarry near the village of Sukhochevo; 3—Moscow region, Shatura district, sand quarry on the outskirts of the town of Roshal.

**Figure 2:**
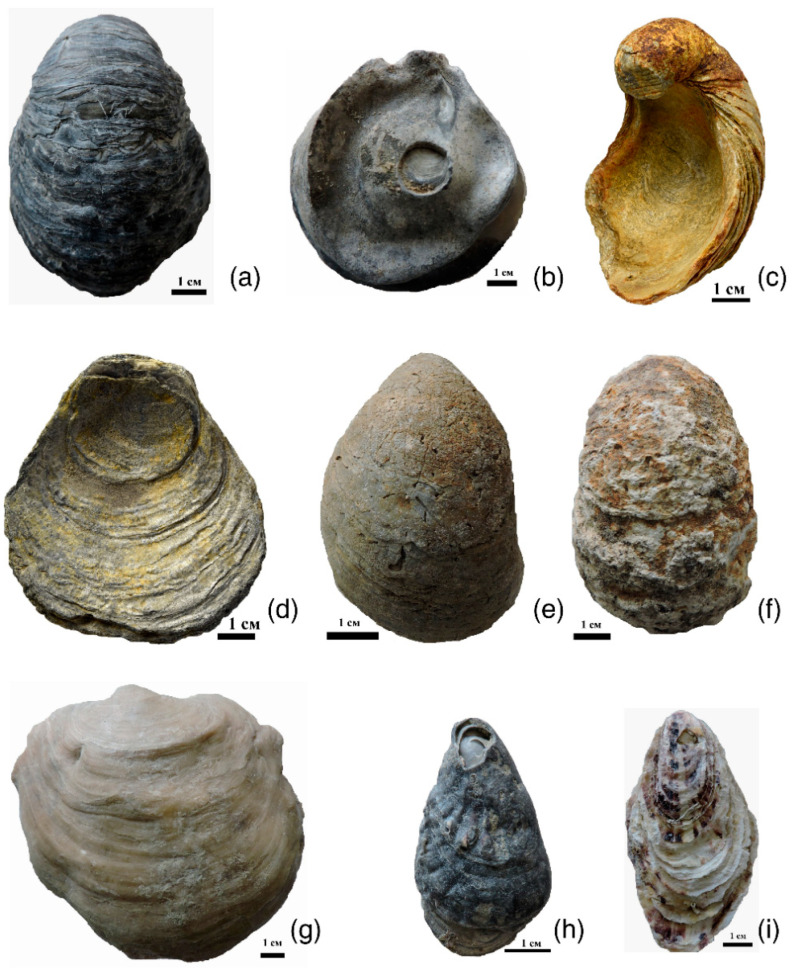
Valves of studied *Gryphaea dilatata* Sowerby, 1816: (**a**) left valve, (**b**) right valve, Kursk region, near Zheleznogorsk, Mikhailovsky quarry, Middle Jurassic, Middle–Upper Callovian; (**c**) left valve, (**d**) right valve Oryol region, Kromy district, sand quarry near the village of Sukhochevo, Middle Jurassic, Middle Callovian; (**e**) left valve, Moscow region, Shatura district, sand quarry on the outskirts of the town of Roshal, Middle Jurassic, Callovian–Upper Jurassic, Lower Oxfordian; (**f**) left valve with a white friable layer on the surface, Oryol region, Kromy district, sand quarry near the village of Sukhochevo, Middle Jurassic, Middle Callovian; (**g**) left valve of *Pycnodonte mirabilis* Rousseau, 1842, Crimea Peninsula, Upper Cretaceous, Maastrichtian; (**h**) right valve of *Ostrea edulis* Linnaeus, 1758, the coast of the Arabatsky Gulf of the Azov Sea in the town of Shchelkino (Crimea Peninsula), Pleistocene, Karangat deposits; (**i**) right valve of *Ostrea edulis* Linnaeus, 1758, coast near the village of Maly Utrish, Black Sea, recent.

Figure 1 displays the locations of the collected samples. Valves from the Mikhailovsky quarry near Zheleznogorsk Town (Kursk region, Russia) from the deposits of the Callovian stage of the Middle Jurassic were studied. The valves collected from the quarry near the village of Sukhochevo (Oryol region, Russia) are also from Callovian deposits. Only left valves of this type were found in the urban quarry of Roshal Town (Moscow region, Russia) (Figure 2a–f). All valves were easily extracted, so they did not need to be cleaned. The complex of accompanying gryphaea fauna was analyzed to determine the geologic age.

To compare with gryphaeas, the left valve of *Pycnodonte mirabilis* Rousseau, 1842, was found (Figure 2g). The genus, *Pycnodonte* Fischer von Waldheim, 1835, also belongs to the family, Gryphaeidae [1]. The valve comes from the Upper Cretaceous, the Maastrichtian deposits of the Crimea Peninsula. A comparison with recent (coast near the village of Maly Utrish, Black Sea; Portugal, port of Lagos) and fossil oysters (coast of the Arabat Bay of the Azov Sea near the town of Shchelkino, Crimea Peninsula; Chushka Spit, coast of the Taman Peninsula—Pleistocene, Karangatian layers) of *O. edulis* was carried out according to a previously published paper [18] (Figure 2h,i).

The valves were glued to glass pins with a special two-component adhesive. The pole figures presented in this paper were measured at the Frank Laboratory of Neutron Physics of the Joint Institute for Nuclear Research (Dubna, Russia). The SKAT texture diffractometer located on channel 7-A of the IBR-2 pulsed nuclear reactor was used [5]. The seventh channel has a long flight base (more than 100 m long), which leads to the good spectral resolution of SKAT. Due to the pulse nature of the neutron flux, the diffractometer implements a time-of-flight measurement technique. SKAT consists of a detector ring (diameter of 2 m) on which 19 detector-collimator complexes are located at the same scattering angle of 2*θ* = 90°.

**Figure 3:**
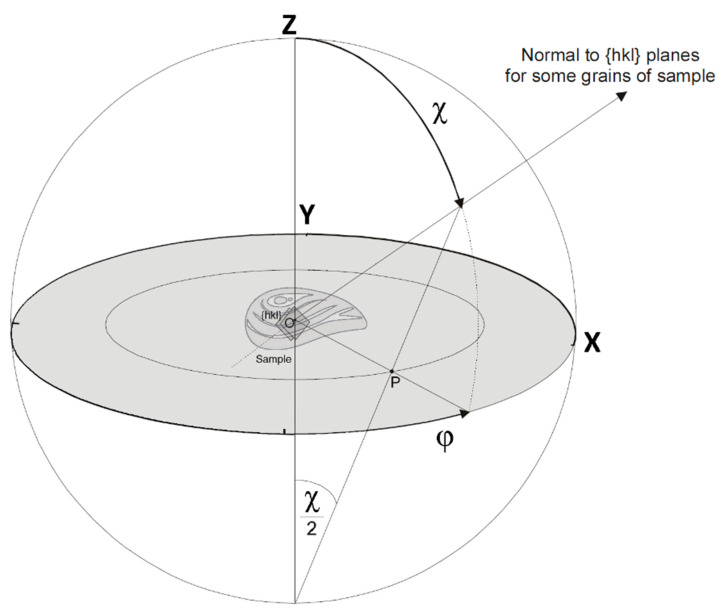
Pole figure construction using stereographic projection. XYZ is a Cartesian coordinate system. χ is the polar angle of a direction in the space, whereas φ is the azimuth angle. Point P is the stereographic projection of a point from the northern hemisphere.

A sample is rotated 360° with a step of 5° about the horizontal axis with an angle of 45° with respect to the incident neutron beam. Rotation is carried out using a goniometer. Thus, 1368 diffraction patterns are recorded during each sample measurement. It should be noted that due to the time-of-flight technique, the pole figures of all minerals (phases) present in the sample are measured simultaneously, i.e., extracted from the same patterns. A neutron beam cross section of 50 × 90 mm makes it possible to measure large samples of up to 100 cm^3^. Such experimental conditions provide the following advantages: measurements at the same scattering angles lead to the same position of the same diffraction reflections for all detectors, which allows us to avoid corrections. Moreover, since the detector modules are located on the ring at angles of 0° to 180°, one rotation about the horizontal axis of the goniometer is sufficient to measure complete pole figures. One more advantage of neutron texture measurements is that the sample surface does not need to be prepared in a special way. This is due to the results being influenced the whole volume of a sample—and not just the surface—because of the high penetration power of neutrons. The most intense, non-overlapped diffraction reflections from each of the recorded patterns were analyzed using the Pole Figure Extractor program [19] to determine the distribution of the corresponding crystallographic planes of the valves. The intensity of one reflection, corresponding to a crystallographic plane with certain Miller indices, gives one point on the pole figure, which is indicated by these indices. To extract the pole figures, we used an approach based on the approximation of diffraction reflections by a bell-shaped distribution, since the signal-to-background ratio was quite high [20]. All pole figures were normalized and smoothed with the same parameter [21]. The most intense diffraction reflections that correspond to crystallographic planes with Miller indices (0006) and (10–14) for calcite were analyzed. The more ordered the mineral crystals were, the higher the intensity was on the pole figure (pole density), which is expressed in units of isotropic orientation distribution (multiple random distribution, mrd). An increase in pole figure intensity is interpreted as texture intensification. When the value of the pole density is equal to 1, it means that the corresponding crystallographic planes are uniformly distributed in the sample in all directions. The analysis was carried out according to the interpretation of the maximum sharpness and the isolines pattern of the pole figures with the Miller indices, (0006) and (10–14). The pole figures are presented by stereographic projections. The stereographic projection is obtained from the spherical one by means of projecting the sphere point onto the equatorial plane. The position of a given pole on the sphere is commonly characterized in terms of two angles. The angle χ describes the inclination of the pole, where χ = 0° is the north pole of the unit sphere and the angle φ characterizes the rotation of the pole, as shown in Figure 3.

To project a point from the northern hemisphere, it should be connected by a ray with the south pole. The intersection of the projecting ray with the projection plane gives the stereographic projection of this point (point P in Figure 3). The pole figures are usually collected by varying the angle of rotation *φ* and the tilt angle χ, where φ and χ are varied from 0–360° and 0–90°, respectively. Thus, the intensity of a particular Bragg reflection measured under varying sample orientations yields an intensity distribution I (hkl) as a function of the χ and φ angles over a sphere, which defines the crystallographic orientation distribution of the grains in the shell. This way, the pole figure represents a variation of the pole density for a selected set of crystallographic planes.

The shell microstructure was recorded using Tescan Vega 2 (Czech Republic, Brno) and CamScan-4 (Cambridge, UK) scanning electron microscopes (Borissiak Paleontological Institute, Russian Academy of Science, PIN RAS). Belemnite rostra was scanned with gold spraying, whereas the bivalve mollusks were scanned without spraying in a low vacuum. We used the results of the X-ray microtomography of ferruginous belemnite rosters from the Upper Jurassic deposits of the Volgian stage (Kuntsevo-Filyevsky Park, Moscow, Russia), using a Skyscan 1172 microtomograph (Belgium, Kontich) (PIN RAS), to demonstrate the effect of iron minerals on calcite. The measurements were carried out with a resolution of 3–30 μm, current of 100 mA, voltage of 103–104 kV, rotation angle of 0.7°, rotation by 180°, frame averaging of 8, random movement of 10, and filter Al (1 mm). The NRecon program, version 1.6.4.1 (Bruker, Belgium, Kontich) was used for visualization.

The photographs for Figure 2c,d were taken by S.V. Bagirov (PIN RAS), and the rest were by A.V. Pakhnevich.

## 3. Results

### 3.1. Determination of the Shells’ Geologic Age

The species, *G. dilatata,* were found in the Middle and Upper Callovian and Lower Oxfordian deposits of the Central part of European Russia [22]. Information about the geologic age of the valves was obtained from fauna assemblages collected together with *G. dilatata,* and from the literature [22,23]. The associated fauna was not extracted along with the valves collected in the Mikhailovsky quarry (Kursk region), so their age was determined according to the data from the book [22]. In this locality, *G. dilatata* are found in clay deposits above the Lower Callovian, that is, they may belong to either the Middle or Upper Callovian layers. Together with mollusk shells, the following fauna complex was found in the locality near the village of Sukhochevo, namely bivalves such as *G. lucerna* Trautschold, 1862; *G. russiensis* Gerasimov, 1984; *Nanogyra nana* Sowerby, 1822; *Pholadomya murchisoni* Sowerby, 1827; *Deltoideum hemideltoideum* Lahusen, 1883, *Goniomya* sp.; traces of vital activity from drilling bivalve, *Lithophaga antiquissima* Eichwald, 1860; rare fragmentary small rostra of belemnites *Cylindroteuthis okensis* Nikitin, 1885; and fragments of ammonite shells from *Erymnoceras* sp.; *Indosphinctes mutatus* Trautschold, 1862; *Hecticoceras* sp. (determined from a gryphaea imprint); and *Kosmoceras* sp. (one of the specimens was identified by a gryphaea imprint). There are numerous drillings on the gryphaea shells. There were also fragments of charred, silicified, and pyritized wood. Due to the presence of species such as *P. murchisoni*, *I. mutatus*, *Erymnoceras* sp., *D. hemideltoideum* and *C. okensis*, the Middle Callovian age (Middle Jurassic) was determined.

The complex of fauna and flora from the location on the outskirts of the town of Roshal is different. It contains trace fossils (burrow filling); fragments of shells and internal casts; imprints of ammonites, of which only *Amoeboceras* sp. has been identified; rare tubes of annelids, such as Serpulidae; single teeth from sharks, such as *Sphenodus stschurowskii* Kiprijanoff, 1880; numerous shells from scaphopod mollusks, *Laevidentalium gladiolus* Eichwald, 1846; silicified fragments from crinoid stems (probably redeposited from Carboniferous); numerous drillings of gryphaea shells, among which there are barnacles’ drillings; bivalves such as *Astarte cordata* Trautschold, 1861; *Cosmetodon* sp.; *Astarte* sp. cf. *A. duboisiana* d‘Orbigny, 1845; *A*. cf. *panderi* Rouillier, 1847 (as well as numerous unidentifiable shell fragments belonging to other species); gastropods such as *Actaeon frearsiana* d‘Orbigny, 1845; *Bourgetia reticulata* Gerasimov, 1992; *Oonia calypso* d‘Orbigny, 1850; belemnites *C. okensis*; *C. oweni* Pratt, 1844; *C. subextensa* Nikitin, 1884; *Hibolites hastatus* Blainville, 1827; *Hibolites* sp.; and wood fragments. Most likely, the fossil remains of the Callovian (Middle Jurassic) and Oxfordian (Upper Jurassic) are mixed in the deposit, possibly even with Volgian forms (Upper Jurassic), but this is unconfirmed data since the finding of the Volgian *Astarte* sp. cf. *A. duboisiana* and *A*. cf. *panderi* does not inspire complete confidence. Species such as *A. cordata*, *Ac. frearsiana*, and *Oo. calypso* are Callovian–Oxfordian [22,23]. However, there are also pure Callovian species, for example, *B. reticulata*, *C. okensis*, *C. oweni*, *C. subextensa*, and *H. hastatus*, as well as pure Oxfordian ones, for example, *S. stschurowskii* and *La. gladiolus*. It is highly likely that the gryphaeas are of early Oxfordian age, since the fauna found in the Oxfordian deposits predominates the exclusively Callovian deposits. Therefore, the geologic age of *G. dilatata* from the Mikhailovsky quarry, which is near the town of Roshal, and the ones near the village of Sukhochevo may not coincide.

### 3.2. Analysis of the Diagenesis Features

The thicknesses of sediments located above the layers with gryphaea shells differ. It is about 10 m in the Roshal quarry, about 20 m in the Sukhochevo quarry, and at least 25 m in the Mikhailovsky quarry. The rocks where the shells of the mollusks were deposited also differ. They were found in bluish-gray clays in the Mikhailovsky quarry, whereas in the Sukhochevo quarry, they were found in dense layers of ferruginous sand—less often in gray clays—and in loose sands in the Roshalsky quarry.

Given this, in different localities, the pressure of the overlying sediments, the characteristics of the host rock, and the degree of shell ferrugination differ, which, as will be shown below using the example of belemnites, can be of great importance for the preservation of the shell material.

The valves of *G. dilatata* were fossilized under various conditions. This can be seen from the preservation features of fossil material. Furthermore, the gryphaea valves from the Mikhailovsky quarry are well preserved. There are almost no drillings of organisms that could be produced not only in vivo, but also posthumously. The shells are dark grey, and small crusts of pyrite (FeS_2_) have been observed on the outer surface.

The valves of the gryphaeas have a light-gray color in the location near the village of Sukhochevo. They contain many perforations, the largest of which belong to the bivalve mollusks, *L. antiquissima*. On the surface of the valves and inside the shell substance, there are traces of ferruginization—sometimes very significant. In this case, the valves become almost completely reddish in color. Some valves are covered with a friable carbonate crust, the origin of which is discussed below (Figure 2f). Possibly, this is one of the surface friable shell layers that was destroyed posthumously.

The valves of the gryphaeas are also light gray in the locality near the town of Roshal. They also have traces of drilling, and in particular, barnacles. Signs of ferruginization are also observed, and some valves have a yellowish-red color or spots on the surface. However, the degree of ferruginization is less than in the locality near the village of Sukhochevo.

### 3.3. Features of Crystallographic Texture

Only calcite is found in all shells. Other minerals/impurities, including iron minerals—for example, pyrite—are contained in small amounts that do not exceed the resolution of the method. We characterized the crystallographic texture through two measured pole figures for each sample. A pole figure is a two-dimensional function on a unit sphere. For crystallographic texture comparison, we used the maximum values on a pole figure with (0006) and (10–14) Miller indices. Moreover, we also analyzed a pole figures’ isoline pattern. As soon as we determined that *G. dilatata* valves could be either convex or flattened, we compared them separately to exclude the sample shape influence.

**Figure 4:**
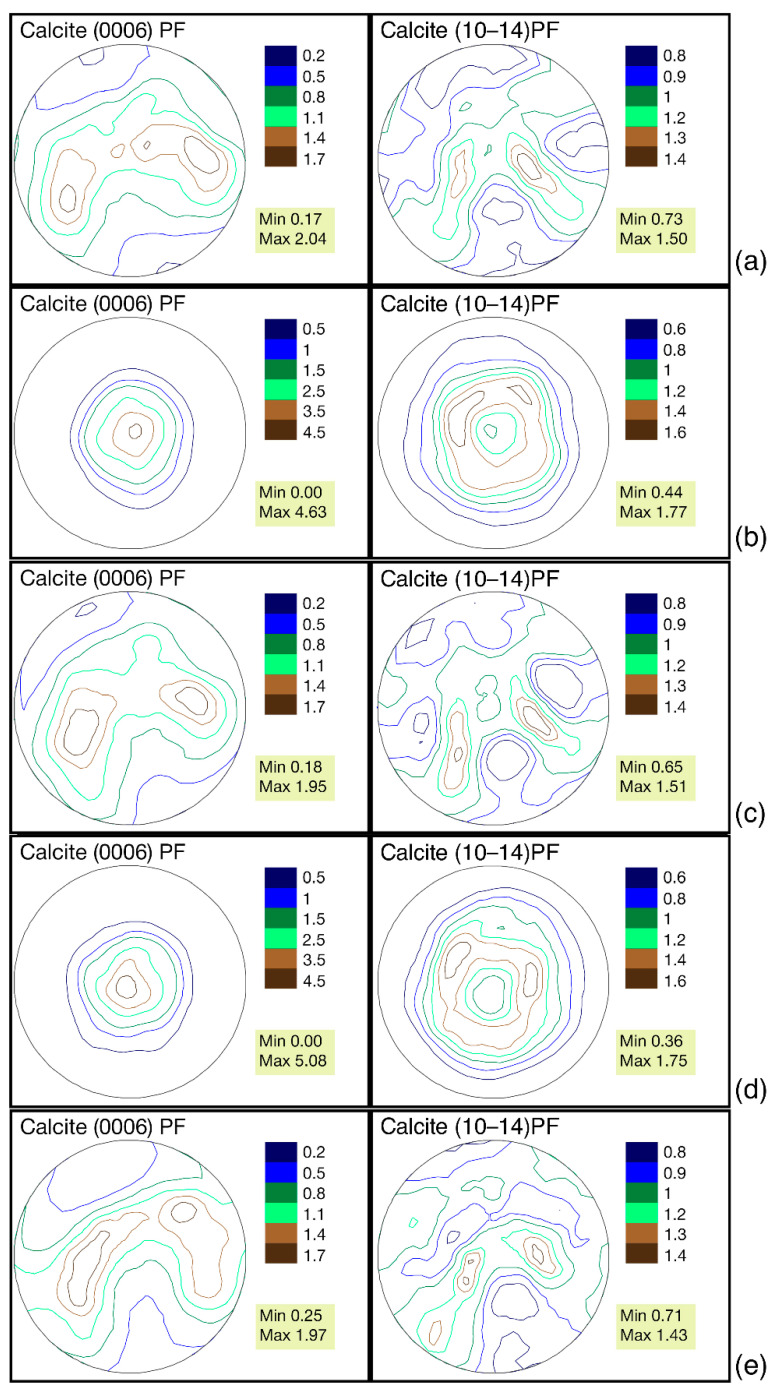
Pole figures of *Gryphaea dilatata* Sowerby, 1816, from different locations: (**a**) Calcite pole figures of the left valve of *Gryphaea dilatata* Sowerby, 1816, Kursk region, near town of Zheleznogorsk, Mikhailovsky quarry, Middle Jurassic, Middle–Upper Callovian; (**b**) Calcite pole figures of the right valve of *Gryphaea dilatata* Sowerby, 1816, Same; (**c**) Calcite pole figures of the left valve of *Gryphaea dilatata* Sowerby, 1816, Oryol region, Kromy district, sand quarry near the village of Sukhochevo, Middle Jurassic, Middle Callovian; (**d**) Calcite pole figures of the right valve of *Gryphaea dilatata* Sowerby, 1816, Same; (**e**) Calcite pole figures of the left valve of *Gryphaea dilatata* Sowerby, 1816, Moscow region, Shatura district, sand quarry on the outskirts of the town of Roshal, Middle Jurassic, Callovian–Upper Jurassic, Lower Oxfordian.

The sharpest crystallographic texture is observed in *G. dilatata* from the Zheleznogorsk quarry, 2.04 mrd, for the convex left valve of shell. In other cases, it varies from 1.95–1.97 mrd. The differences in these values are minimal. We have previously established [18] that for the mussels, *Mytilus galloprovincialis* Lamarck, 1819, it varies within 1.03 mrd, depending on the habitat. Therefore, these values seem insignificant. The values of the pole density sharpness on the pole figure (10–14) are smaller; they vary from 1.43–1.51 mrd. At the same time, the highest value was revealed for the shells collected in the quarry near the village of Sukhochevo. Furthermore, in this case, the range of variation is very small (Figure 4).

**Figure 5:**
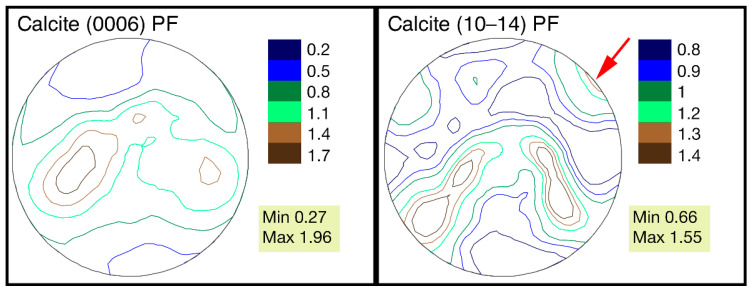
Calcite pole figures for the left valve of *Gryphaea dilatata* Sowerby, 1816, with a friable white surface layer, Oryol region, Kromy district, sand quarry near the village of Sukhochevo.

From the point of view of the isoline distribution, the pole figures can be characterized as follows. The distribution of the pole density for both pole figures has a horseshoe shape with two centers of greatest sharpness. There are isolines with a minimal pole density almost at the edges of the pole figure, (0006). On pole figure (10–14), similar isolines are located on the sides. A similar pattern is observed in valves from all three localities (Figure 4). Separately, it is necessary to consider the left valve of *G. dilatata* with a friable carbonate layer on the outer surface from the quarry near the village of Sukhochevo. The maximal sharpness of the pole density is 1.96 mrd (pole figure (0006)) and 1.55 mrd (pole figure (10–14)). In the first case, this falls within the considered range for other valves; in the second case, it slightly exceeds the maximum value by 0.05 mrd (Figure 5).

The red arrow shows the area of anomalous sharpness maxima.

**Figure 6:**
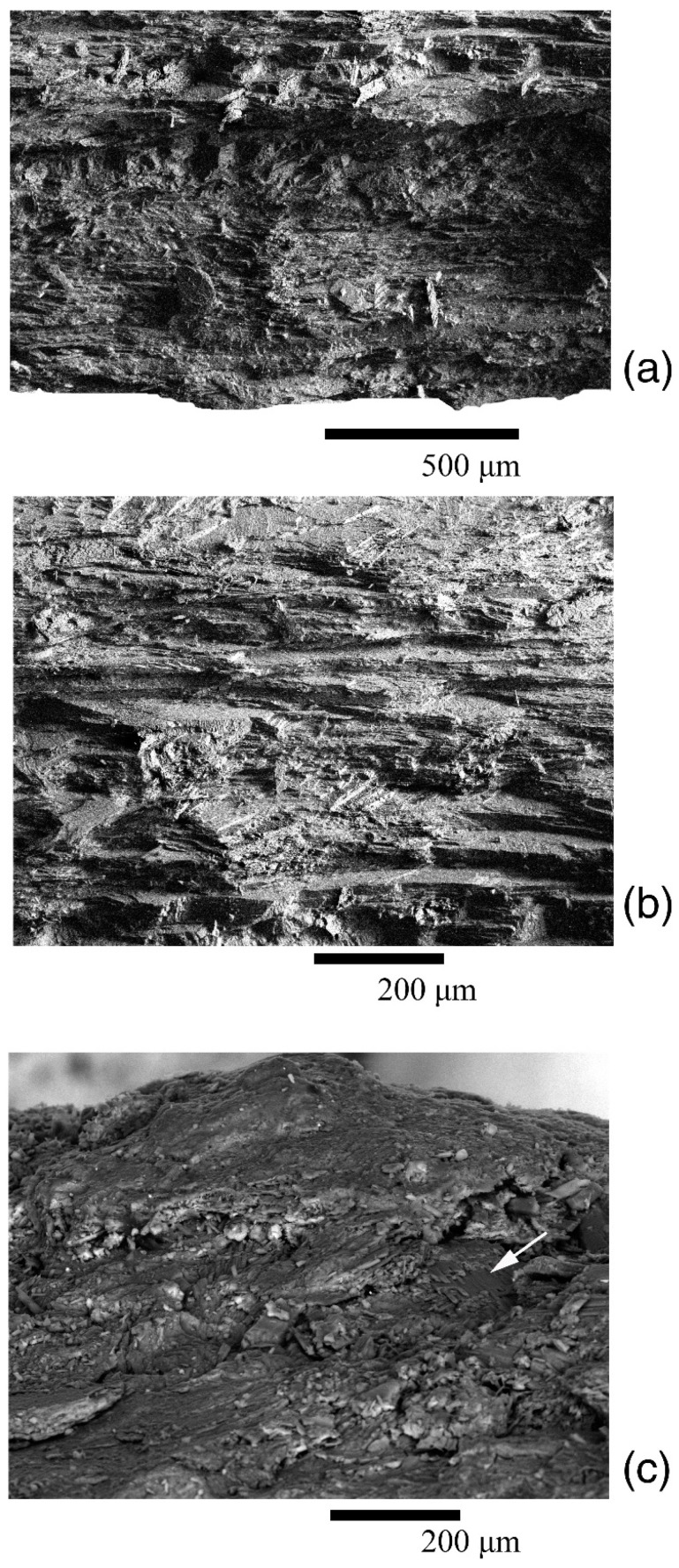
Microstructure of valves of the *Gryphaea dilatata* Sowerby, 1816: (**a**,**b**) split of the left valve; (**c**) friable white layer, the white arrow shows the elements of the microstructure, Oryol region, Kromy district, sand quarry near the village of Sukhochevo, Middle Jurassic, Middle Callovian.

The distribution of the pole density on pole figure (0006) also has a horseshoe shape, but with three centers of greatest sharpness—one of them being more pronounced than the others. Differences are also noted for pole figure (10–14), namely a small maximum with coordinates (χ = 90°, φ = 45°) is present at the edge of the pole figure, which was not observed for other valves. The microstructure of gryphaeas valves is foliate (Figure 6a,b), and the plates are composed of foliated calcite crystals, as was shown previously [24,25].

When analyzing the outer layer using an electron microscope, it was revealed (Figure 6c) that the friable substance contains elements of the microstructure in the form of separate and grouped plates. There are also clusters of irregularly-shaped globules. The rest of the substance is a continuous mass without any order of elements. Since no fundamental differences are found in the crystallographic texture of the valves, the friable layer on the valve surface may refer to shell material, which was possibly modified to some extent. However, it is absent for most shells; this layer was probably destroyed posthumously. The absence of foulers on the surface of all shells is associated with this fact, although the valves of these mollusks could potentially be a good place for various organisms to settle. During the diagenesis process, the layer where the foulers were located collapsed, and there were not even traces of their presence. However, there are drillings from organisms that could extend to different depths of the shell material, including the inner valve surfaces. Moreover, drillers could perform them after the mollusk’s death.

The pole figures of (10–14) for the *G. dilatata* valve with a friable carbonate layer from the Sukhochevo quarry and the valve from the Roshal quarry are very similar in their isoline patterns. However, we confirmed the presence of valve recrystallization by the SEM method in the first case, whereas we can only assume this in the second case.

A different pattern of crystallographic texture was observed for slightly-concave right valves from two localities: the Zheleznogorsk quarry and the quarry near the village of Sukhochevo (Figure 2b,d and Figure 4b,d). The texture sharpness for pole figure (0006) varies from 4.63–5.08 mrd, whereas for pole figure (10–14), it varies between 1.75–1.77 mrd. That is, both values are higher than those of strongly-convex left valves. Given this, the crystals from the right valves are more ordered. This can also be seen by the isoline distributions on the pole figures. The center of the pole density maxima is localized close to the central parts of the pole figures. Most of the isolines are circular. The center of the pole density maxima is bifurcated on pole figure (10–14), and the crystal distribution is very dependent on the valve shape. Given this, it was probably easier for mollusks to arrange calcite crystals in a thick, almost flat, valve than in a thick, strongly-convex one.

**Figure 7:**
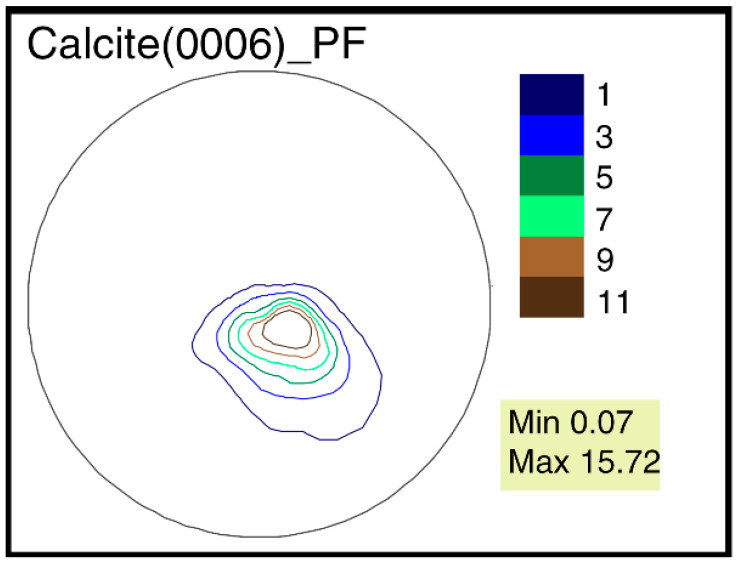
Calcite pole figure (0006) of the valves of *Mytilus galloprovincialis* Lamarck, 1819, Tuzla Spit, Kerch Strait, Pleistocene, Karangat deposits.

A similar pole figure (0006) is known for mussels of the genus, *Mytilus* Linnaeus, 1758. This pole figure looks like deformed circles inscribed in each other, which are located offset from the center. However, its sharpness is much higher, up to 15.72 mrd, for Pleistocene representatives of the species, *M. galloprovincialis* [17] (Figure 7).

**Figure 8:**
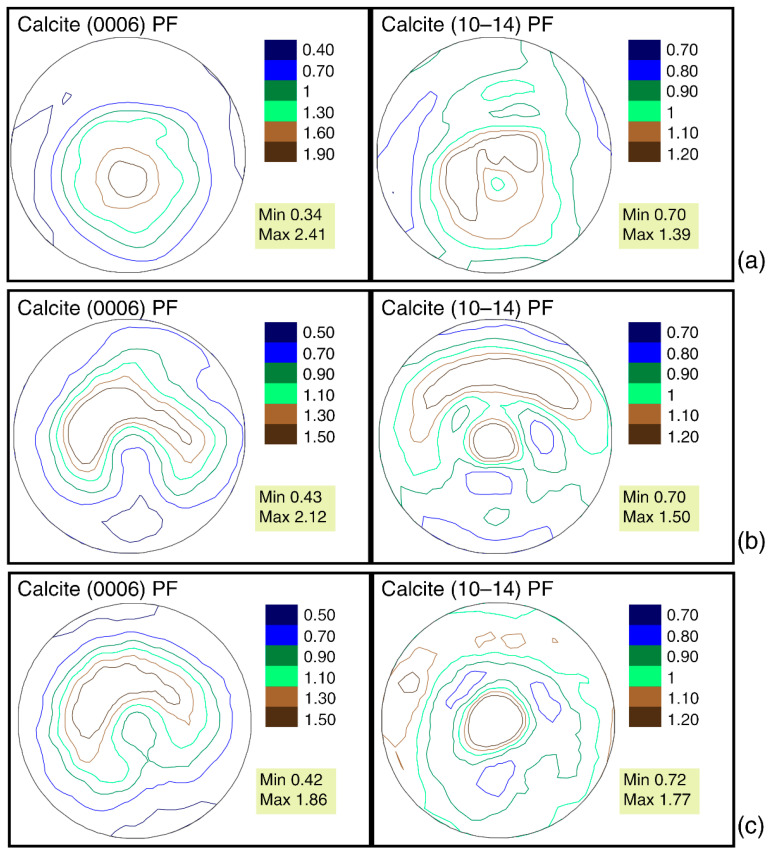
Calcite pole figures of thick-walled valves from fossil bivalves: (**a**) left valve of *Pycnodonte mirabilis* Rousseau, 1842, Crimea Peninsula, Upper Cretaceous, Maastrichtian; (**b**) right valves of *Ostrea edulis* Linnaeus, 1758, the coast of the Arabatsky Gulf of the Azov Sea in the town of Shchelkino (Crimea Peninsula), Pleistocene, Karangat deposits; (**c**) right valves of *Ostrea edulis* Linnaeus, 1758, Taman Peninsula, Chushka Spit, Pleistocene, Karangat deposits.

It is interesting to compare the crystallographic texture of studied *G. dilatata* valves with the texture of thick valves from closely-related mollusks. To do this, recent mollusks, such as *O. edulis,* from the close family, Ostreidae, and the left valve of the mollusk, *P. mirabilis—* family Gryphaeidae—were taken from the Maastrichtian deposits of the Crimea Peninsula. The left, thick-walled valve of *P. mirabilis* was studied. It is convex in shape (Figure 2g), but not as strongly as the left valves of *G. dilatata.* The shell contains only calcite. Its pole figure (0006) (Figure 8a) is very similar to figure (0006) of the almost-flat right valves of *G. dilatata,* and the calcite crystals are less ordered. The pole density maximum of pole figure (0006) of the *P. mirabilis* left valve is 2.41 mrd, while this value varies for the right *G. dilatata* valves—within 4.63–5.08 mrd. The isoline pattern of pole figure (10–14) is similar to that of the same pole figure of *G. dilatata* right valves. The pole density maximum is 1.39 mrd, which is less than in the left valves of *G. dilatata.* Moreover, the pole density sharpness of both *P. mirabilis* pole figures are much less than one in the right valves of *G. dilatata*, although the pole figures have similarities. Thus, with an increase in the convexity of the thick-walled valves, the sharpness of the crystallographic texture decreases. The pole figures of the left valves differ significantly, although the values of the greatest sharpness either coincide, or are very close, for related genera from the same family.

**Figure 9:**
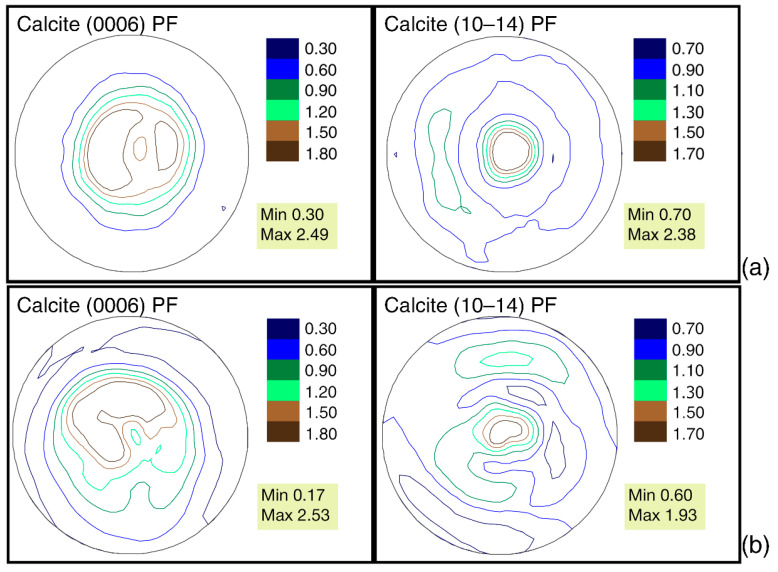
Calcite pole figures of the thick-walled valves of *Ostrea edulis* Linnaeus, 1758: (**a**) right valve, coast near the village of Maly Utrish, Black Sea; (**b**) left valve, Portugal, coast near the port of Lagos.

**Figure 10:**
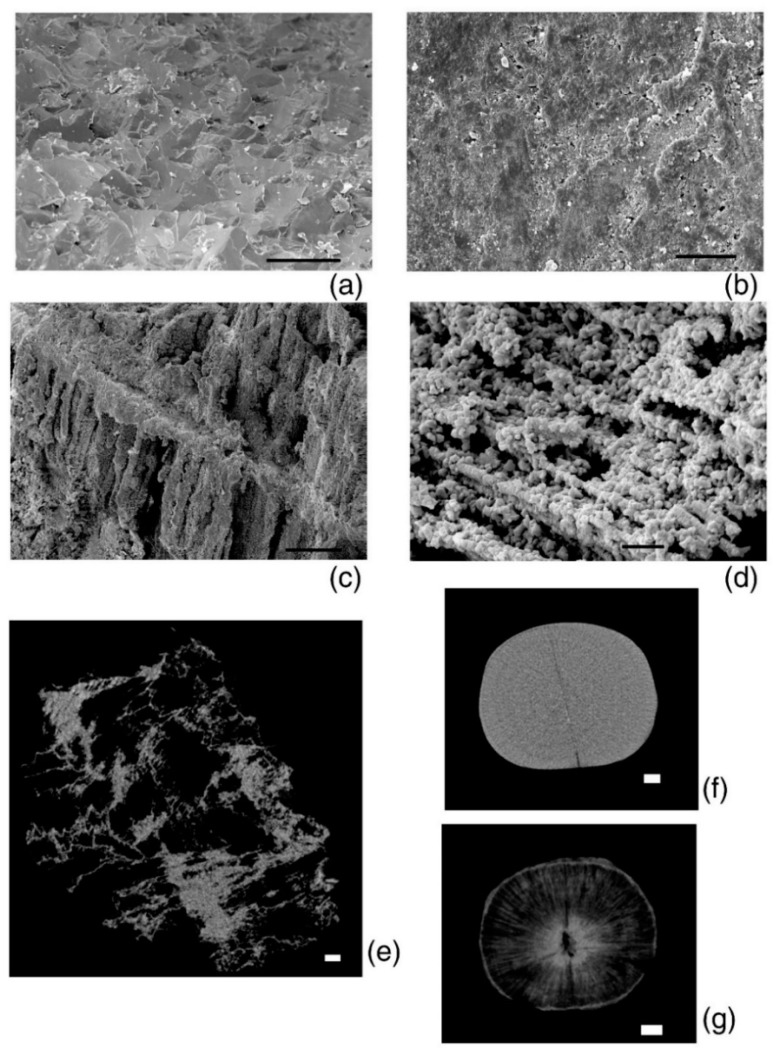
Surface and internal microstructure of belemnite rosters (Upper Jurassic, Upper Volgian; Moscow, Kuntsevo–Filyevsky Park) substituted and unsubstituted with iron minerals: (**a**) transverse split of belemnite rostra not substituted with iron minerals, scale is 100 µm; (**b**) surface of belemnite rostra not substituted with iron minerals, scale is 30 µm; (**c**,**d**) microstructure of the rostrum substituted with iron minerals, scales are 100 and 10 µm, respectively; (**e**) virtual section of a growth fragment substituted with iron minerals, X-ray microtomography, almost all calcite structures are destroyed inside, scale is 100 µm; (**f**) transverse virtual section of a belemnite roster not substituted with iron minerals, X-ray microtomography, scale is 1 mm; (**g**) transverse virtual section of the belemnite rostrum substituted with iron minerals, X-ray microtomography, scale is 1 mm.

The shells of recent oysters also consist of calcite and have a variable, flattened, or convex shape with various elements of radial and concentric sculpturing on the shell’s surface. The maximum values of pole figure (0006) of the recent oysters, *O. edulis* (Black Sea, Maly Utrish), right valves are 2.49 mrd (Figure 9a), and 2.38 mrd for pole figure (10–14). For the left valve of *O. edulis* (port Lagos, coast of Portugal), the same values are 2.53 and 1.93 mrd, respectively (Figure 9b). The range of sharpness variation, as in the case of gryphaea, is small. For recent oysters, the sharpness values are slightly higher than the ones in the left valves of *G. dilatata,* and significantly lower than the same values for pole figure (0006) of the flat right valves of gryphaeas. For fossil oysters (right valves) of the same species from Pleistocene deposits, the maximum sharpness of pole figure (0006) varies in the range of 1.86–2.12 mrd, while that of pole figure (10–14) varies from 1.5–1.77 mrd (Figure 8b,c). The ranges of variation are also small. The maximal pole figure values are less than those of recent oysters, which has already been noted [18]. The largest sharpness values of the pole figure (0006) for *G. dilatata* left valves (Figure 4) and the Pleistocene *O. edulis* right valves (Figure 8b,c) are almost the same, and for pole figure (10–14) of *G. dilatata* they are slightly lower, although the largest value is smaller than the lower ones of the range for the fossil *O. edulis.* A comparison of the right and left valves of oysters is justified, given that the left and right valves of *O. edulis* differ a little in shape and sculpture. The isoline patterns on the pole figures of both valves are also slightly different.

The calcite pole figures (0006) of the left and right valves of recent oysters remained an arc according to the isoline pattern (Figure 9), but with different curvature degrees. In this way, they are similar to the ones for the pole figures of the *G. dilatata* left valves. However, the latter ones have a much stronger isoline curvature on the pole figure’s central part. 

The calcite pole figure (0006) of recent oysters from the coast near the village of Maly Utrish is very close to the same pole figure of the *G. dilatata* right valve and the *P. mirabilis* left valve. The isolines of this pole figure have an almost circle shape, but the central part is divided into three unequal sectors, the larger of which is depressed, which gives it the shape of a wide, short arc. The calcite pole figure (10–14) of oysters has several circles or ovals inscribed into each other in the central part (Figure 9).

## 4. Discussion

The Jurassic *G. dilatata* bivalves turned out to be a very good object for studying the influence of habitat and fossilization conditions on their calcite crystallographic texture. The studied shells were found at a distance of tens (Mikhailovsky and Sukhochevo quarries) and hundreds (Roshal and Sukhochevo, Mikhailovsky and Roshal quarries) of kilometers from each other, that is, the mollusks lived in different water areas under different habitats. *G. dilatata* from the quarries near the village of Sukhochevo and Mikhailovsky could have lived at about the same time, and the mollusks from the quarry near the town of Roshal are slightly younger than the other samples. Since even Callovian *G. dilatata* can come from different substages (Middle and Upper), the habitats at different times are unlikely to be the same. In addition, by the beginning of the Late Jurassic, the sea-bay, which was on the territory of the Russian platform, became more open, connecting with other water areas [26]. This affected the change in habitat for example, resulting in an increase in salinity, or the transition of terrigenous bottom sediments to calc-terrigenous ones. In addition, the presence of a large amount of charred and pyritized wood in the Sukhochevo quarry is evidence of the coastal strip’s proximity, which means an increased drift of terrigenous material into the sea basin.

The degree of ferruginization and calcite replacement with iron minerals differs for grypheas from different localities. This diagenetic factor can significantly affect the microstructure of a fossil, as was observed in the case of belemnite rostra from the Kuntsev–Filyovsky Park in Moscow (Upper Jurassic deposits, Volgian stage) (Figure 10).

Despite this, the pole figures of all studied *G. dilatata* samples are very similar in terms of the isoline pattern, that is, the habitat and fossilization conditions affected the crystallographic texture very little. Moreover, the pole figures of the samples from different places differ very slightly according to the largest texture sharpness values. This is illustrated by two calcite pole figures, (0006) and (10–14). The most variable sharpness values are observed for pole figure (0006), so it is the most significant for the analysis of the different influences on the crystallographic texture.

The only deviation from the general trend is the left valve of the gryphaea with a friable carbonate surface layer. Despite this, the values of the maximum texture sharpness coincide for this valve and the rest of the left ones. However, there are differences in the pole figures’ isoline patterns. An additional peak of maximum sharpness on two pole figures is likely associated with the recrystallization of the valve surface layer, which is also confirmed by microstructure studies. That is, the fact of recrystallization was reflected not in the maximum texture sharpness values, but in the isoline pattern, since the recrystallized layer contains weakly-oriented crystals, and the most strictly-oriented crystals are located in the remaining layers—which all valves have. This is why the values of maximum sharpness are almost the same.

**Figure 11:**
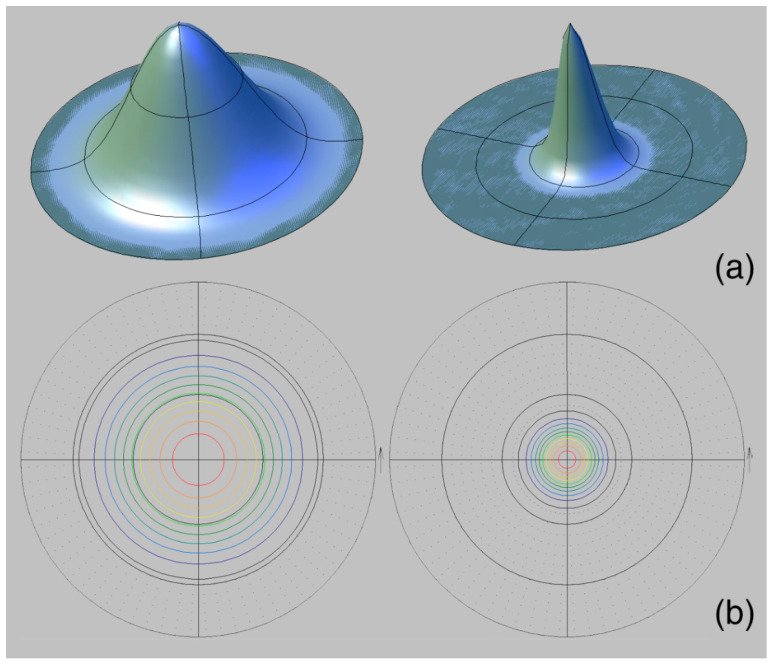
Models of crystal distribution peaks: (**a**) Illustration of the crystallographic texture sharpness; (**b**) its presentation on the pole figures. The circles with different color are isolines with the same pole density values.

Using the gryphaeas, it is possible to observe how different parts of the skeleton—in this case, the valves—of the same organism have significant differences in crystallographic texture. These differences are associated with different valve shapes. They are reflected both in the difference of the pole figures’ isoline patterns, and in the sharpness values. The maximum texture sharpness of the *G. dilatata* right valves is 5.08 mrd. Their pole figures (0006) are very close to axial ones. A similar picture is found for recent and fossil *M. galloprovincialis*. The pole figures (0006) of mussels are almost axial, but the maximum texture sharpness is 15.72 mrd. This situation can also be represented by the example of two peaks: weak and sharp (Figure 11). If one cuts the peaks from their base to the highest point by several sections and looks from above, one gets an axial pole figure, but the cut lines of the different peaks are located at different distances. The crystalline orientations are closer to each other for sharper peaks.

The right valves of gryphaeas, as well as the thick-walled left valves of *P. mirabilis*, have axial pole figures (0006). At the same time, pole figure (10–14) of *P. mirabilis* has an arcuately curved isoline for the maximum of the pole density intensity. A similar situation is observed for oyster’s pole figure (0006). In recent oysters, the pole figure (0006) isoline pattern varies from a figure with an arcuate maximum to an almost axial one. Meanwhile, pole figure (10–14) for most of the studied valves has an axial character with a maximum in the center. Only *G. dilatata* has a curved arcuate center maximum. All studied shells were related, i.e., *G. dilatata* and *P. mirabilis* are members of the same family, while *O. edulis* belongs to the close family, Ostreidae. Other similarities are that their shells are thick-walled, and *G. dilatata* and *P. mirabilis* have convex left valves, while other valves (the right gryphaeas valves and oysters) are flattened. At the same time, for the fossil oysters and the recent ones from the coast of Lagos, pole figure (0006) for the left and right valves has a slightly curved maximum, which is similar to the same pole figure of the left *G. dilatata* valves. Furthermore, only for recent Black Sea *O. edulis* does pole figure (0006) of the right valves represent a transitional variant between the axial pole figure and the one with an arcuately-curved isoline for the maximum of the pole density intensity. In this regard, it can be concluded that in the thick-walled shells of the Gryphaeidae and Ostreidae families’ bivalve mollusks, the pattern of isolines varies from almost axial to a figure with an arcuately-curved isoline for the maximum of the pole density intensity.

## 5. Conclusions

There are few works concerning the study of the global crystallographic texture of fossil objects. They cannot be fully compared with the results of local crystallographic texture studies fulfilled using X-ray or EBSD diffraction due to the fundamental difference in the studied volumes of shell material. Moreover, in the case of X-rays or EBSD, the specimen is a small piece of the valve, which is not easily related to the coordinate system of the whole valve. Studies of the global crystallographic texture of fossilized shells of the same species from different geological layers and locations was never carried out before. Thus, we can assess the degree of influence of diagenetic and paleoecological factors on the crystallographic texture of calcite in Jurassic shells, since we have not previously found a single factor that would affect the change in such a texture for recent and Pleistocene mollusks. As well, the global crystallographic texture of objects subjected to recrystallization has never been evaluated. 

As a result of this study, some important aspects of the calcite crystallographic texture of fossil and recent bivalve mollusk shells have been revealed. Since there are only a few results for whole shell or valve crystallographic texture studies that have been obtained by neutron diffraction, the interpretation of both the numerical values of the pole figures maxima and the isoline pattern is very important.

1. For the first time, the complete pole figures of *G. dilatata* shells were measured using time-of-flight neutron diffraction.
2. The studied *G. dilatata* lived at considerable distances from each other. The mollusks likely did not coincide in their time of habitation. This means that their habitats were different.
3. *G. dilatata* from the three localities almost do not differ in their crystallographic texture maximum sharpness, i.e., the habitat conditions and fossilization did not affect the texture, and it can be considered as a very stable feature.
4. The largest differences of the crystallographic texture sharpness have been noted for the calcite pole figure (0006). Therefore, it is possible to reveal the greatest variability by comparing these figures’ sharpness for different objects.
5. The features of *G. dilatata* valve recrystallization, which affect the crystallographic texture, were revealed for the first time using time-of-flight neutron diffraction. They were reflected in the pole figure isolines, but do not appear in the values of maximum sharpness, since the entire valve was not recrystallized and the pole density was low in the recrystallized part. The maximum texture sharpness was determined by the non-recrystallized part of the valve.
6. It has been established that for one mollusk, the crystallographic texture of the left and right valves of various shapes differs: to a greater extent by the pole figures’ isoline patterns, to a lesser extent by the values of maximum sharpness.
7. The calcite pole figures and the values of the maximum sharpness of the *G. dilatata* and *O. edulis* left valves are similar. The pole figures isoline patterns and values of the pole density maximum of *G. dilatata* right valves are similar, and along the same parameters as the *P. mirabilis* left valve. All of these species are characterized by either an axial pole figure (or close to it) or a figure with an arcuately-curved sharpness maximum. Transitions between them are found for the recent *O. edulis*. It is possible that these pole figures with have isoline patterns that are characteristic of thick-walled shells.
8. The thick-walled valves of *G. dilatata*, *O. edulis*, and *P. mirabilis* have small values of maximum texture sharpness. They are very close for these mollusks. Perhaps, it is a characteristic feature of all thick-walled valves and reflects an adaptation to shell construction.

## Data Availability

The data supporting reported results can be obtained on request from the article authors.
